# Supplementary material for: Dietary Supplementation with Popped Amaranth Modulates the Gut Microbiota in Low Height-for-Age Children: A Nonrandomized Pilot Trial
Source: Foods. 2023 Jul 20;12(14):2760. doi: 10.3390/foods12142760 (PMC10379428; doi:10.3390/foods12142760)
Supplement: Supplementary file 1 [file foods-12-02760-s001.zip › foods-2480845-supplementary.pdf]

SUPPLEMENTARY INFORMATION

Dietary supplementation with popped amaranth modulates the gut microbiota in low height-for-age children: A nonrandomized pilot trial

Supplementary Table S1. Proximal composition of popped amaranth (*Amaranthus hypochondriacus*)

**Amaranth**	**Popped grain**
**Protein**	15.8 ±0.6
**Fat**	6.7 ±1.4
**Fiber**	3.8 ±0.9
**Ashes**	2.2 ±0.3
**Carbohydrates**	63.4 ±3.9

Mean of triplicates ± standard deviation

Supplementary Table S2. Hematic cytometry of participant's children with low-height for age according to HAZ index

Determination	Normal	Undernutrition		Biological limits
	(n = 21)	(n = 9)	(n = 7)	
**Red blood cells**				
Erythrocytes (x 10^6)	4.9 ± 0.1	4.8 ± 0.1	4.7 ± 0.1	4.3 - 5.2
Hemoglobin (g/dL)	13.2 ± 0.1	13.2 ± 0.4	13.6 ±0.3	12.5 – 15
Hematocrit (%)	40.7 ± 0.4	40.9 ± 0.9	**42.4 ± 0.7**	37.5 – 48
Mean corpuscular volume MCV (fL)	83.6 ± 0.8	84.9 ± 0.9	**89.7 ± 1.1**	83 – 100
Corpuscular hemoglobin (pg)	27.1 ± 0.3	27.4 ± 0.6	**28.9 ± 0.4**	27 – 32
Mean corpuscular Hb concentration (g/dL)	32.4 ± 0.2	32.3 ± 0.4	32.0 ± 0.2	32 – 34.5
**Platelets**				
Platelet (miles)	282 ± 15	338 ± 33	277 ± 35	150 - 400
**White blood cells**				
Total Leukocytes (x 10^3)	7.6 ± 0.5	6.7 ± 0.6	6.0 ± 0.4	4.5-13.5
Total Neutrophils (%)	57 ± 2	49 ± 3	48 ± 4	
Segmented (%)	57 ± 2	49 ± 3	48 ± 4	
Band (%)	0	0	0	
Metamyelocytes (%)	0	0	0	
Myelocitos (%)	0	0	0	
Promyelocytes (%)	0	0	0	
Blasts (%)	0	0	0	
Eosinophils (%)	0	0	2 ± 1	
Basophils (%)	0	0	1 ± 0	0-3
Monocytes (%)	3 ± 0	4 ± 0	6 ± 0	2-8
Lymphocytes (%)	40 ± 2	47 ± 3	44 ± 4	20-40

One-way ANOVA was carried out followed by Tukey´s post-hoc test. A *p*-value <0.05 was considered as statistically significant.

Supplementary Table S3. Serum biochemical profile of participant children

Determination	Normal size<br>n = 21	Undernutrition	
		**start<br>n = 9**	**after 3 months<br>n = 7**
Glucose (mg/dL)	87 ±10	82 ±20	95 ±6.5
Triglycerides (mg/dL)	105 ±17	115 ±18	121 ±12
Total cholesterol (mg/dL)	133 ±19	146 ±31	151 ±28
Urea (mg/dL)	23 ±7.9	15.6 ±6.3	24.0 ±9.0
Uric Acid (mg/dL)	3.3 ±0.8	3.1 ±0.4	3.2 ±0.6
Creatinine (mg/dL)	0.7 ±0.1	0.70 ±0.1	0.70 ±0.1
Total protein (g/dL)	8.1 ±0.4	8.5 ±0.3	8.3 ±0.6
Albumin (g/dL)	4.8 ±0.4	5.0 ±0.3	5.2 ±0.6
LDH (U/L)	84 ±13	92 ±12	97 ±13
ALKP (U/L)	298 ±80	301 ±85	347 ±127
AST (U/L)	6.5 ±1.5	6.6 ±0.9	6.9 ±0.6
ALT (U/L)	2.9 ±0.9	2.0 ±0.7	2.6 ±1.9
GGT (U/L)	4.7 ±1.6	5.9 ±1.3	7.6 ±1.9
Total bilirubin (mg/dL)	0.5 ±0.3	0.5 ±0.2	0.6 ±0.2
Direct bilirubin (mg/dL)	0.1 ±0.03	0.1 ±0.03	0.1 ±0.03
Indirect bilirubin (mg/dL)	0.4 ±0.3	0.4 ±0.2	0.5 ±0.2

Data is the mean of triplicates ± standard deviation. *After 3 months of amaranth consumption. One-way ANOVA was carried out followed by Tukey´s post-hoc test. A *p*-value <0.05 was considered as statistically significant.

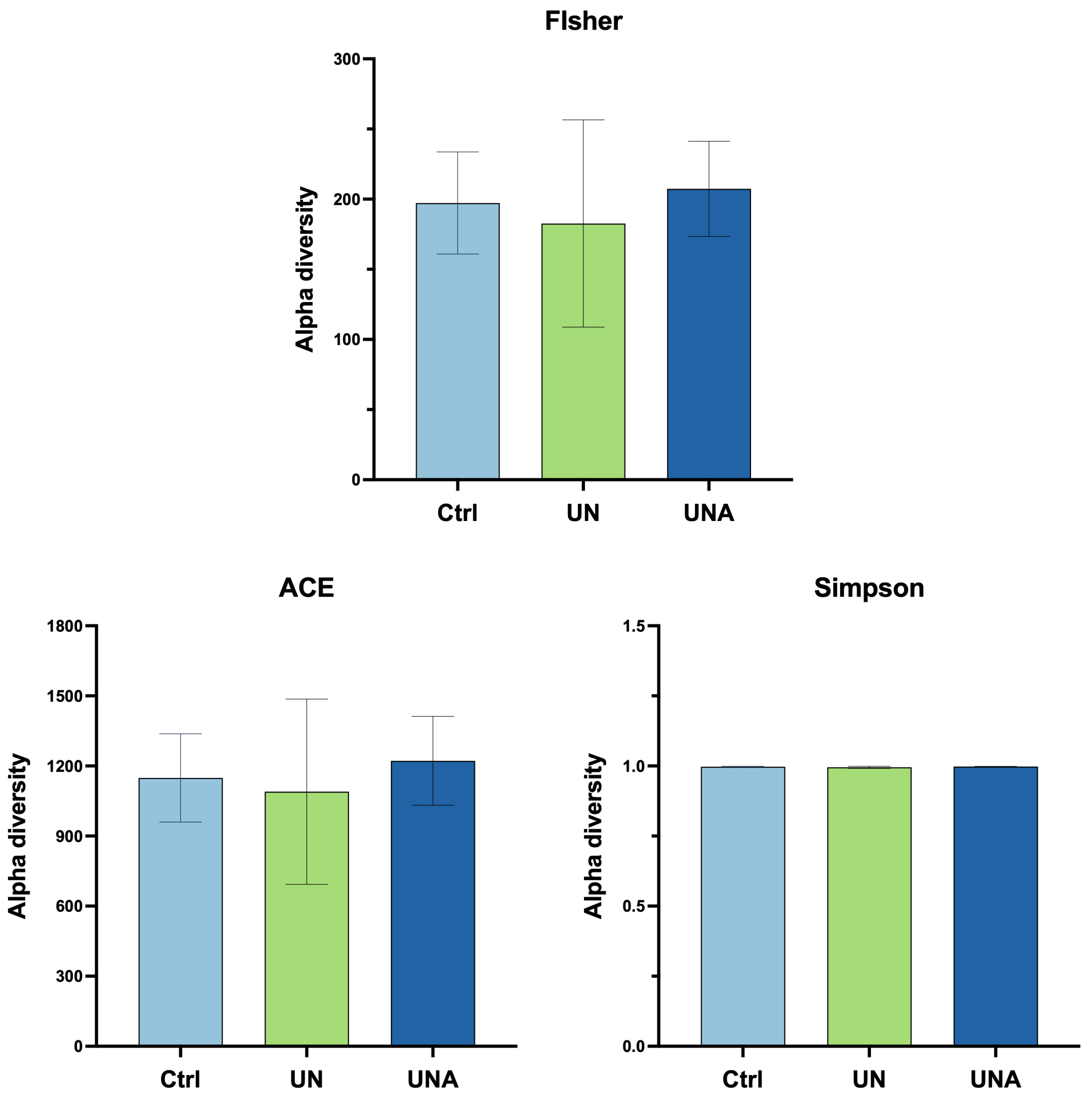

Supplementary Figure S1. Alpha-diversity differences amongst child's groups. Fisher, ACE (abundance-based coverage estimators), and Simpson values. Columns shows the means values and bars the standard deviation. ONE-way ANOVA was carried out followed by Tukey´s post-hoc test at $p < 0.05$. Ctrl=control group of children with normal weight-high for age; UN=undernutrition children; UNA=undernutrition group after three months of amaranth consumption.

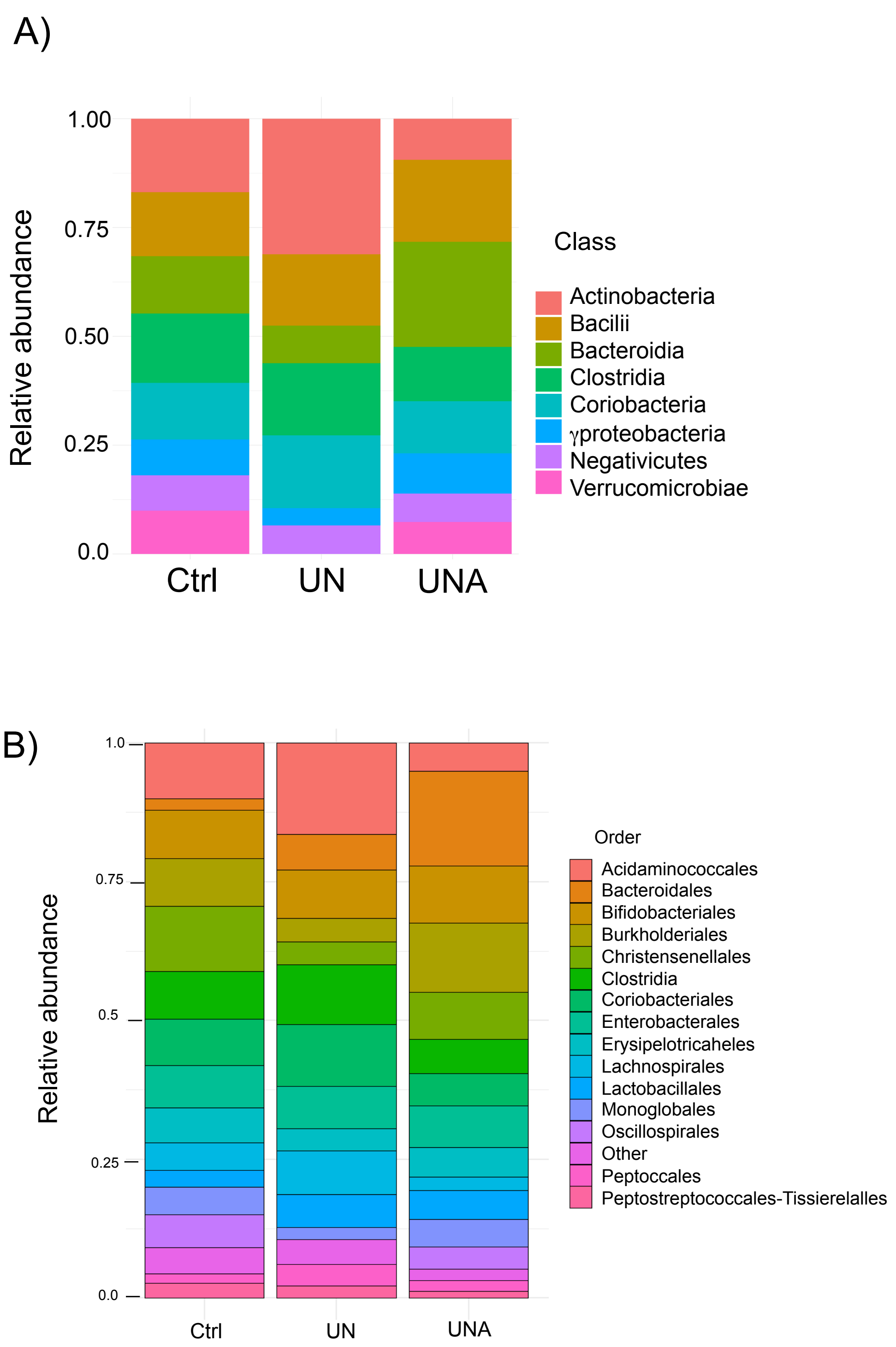

Supplementary Figure S2. Distribution of gut microbiota at A) class and B) order levels. Data showed the top-ten groups in each taxonomy clade.
